# Conjugation prepared by wet-Maillard reactions improves the stability and properties of lutein and lycopene loaded nanoparticles

**DOI:** 10.1007/s13197-024-05976-4

**Published:** 2024-04-07

**Authors:** Tugba Dursun Capar, Hasan Yalcin

**Affiliations:** Food Engineering Department, Engineering Faculty, Erciyes University, Kayseri, Turkey

**Keywords:** Lutein, Lycopene, Encapsulation, Maillard reactions, Stability

## Abstract

In this study, lutein and lycopene were encapsulated in plant protein (faba bean protein concentrate, (FPC))-carrageenan (Car) conjugates prepared by Maillard reaction in an aqueous media. The conjugation improved encapsulation yield that reached to 82.69% and 93.07%, for lycopene and lutein, respectively. The mean particle diameters for lutein loaded nanoparticles observed smaller in FPC-Car conjugates (66.60 nm) than FPC (71.49 nm). Scanning electron microscopy images showed that FPC-Car conjugates were more spherical and no fractures or fissures on the surface, revealing that wall materials provided better protection and retention for core materials. The diameter of lycopene nanoparticles coated with FPC remained constant between pH 3–4 and 7–9 but increased to 220 nm at pH 4–6. Even though the diameter of lutein nanoparticles coated with FPC remains steady between pH 5 and 9, increased to 953 nm at pH 3. The bioaccessibility of the lutein or lycopene samples encapsulated by FPC were found as higher than FPC-Car conjugates. These findings suggest that protein-polysaccharide conjugates could be used as a wall material to encapsulate lipophilic lutein and lycopene in order to improve their stability, property and bioaccessibility. As a result, FPC-Car conjugates may be an alternative for the formation of functional beverages as well as other nutraceutical products.

**Graphical abstract:**

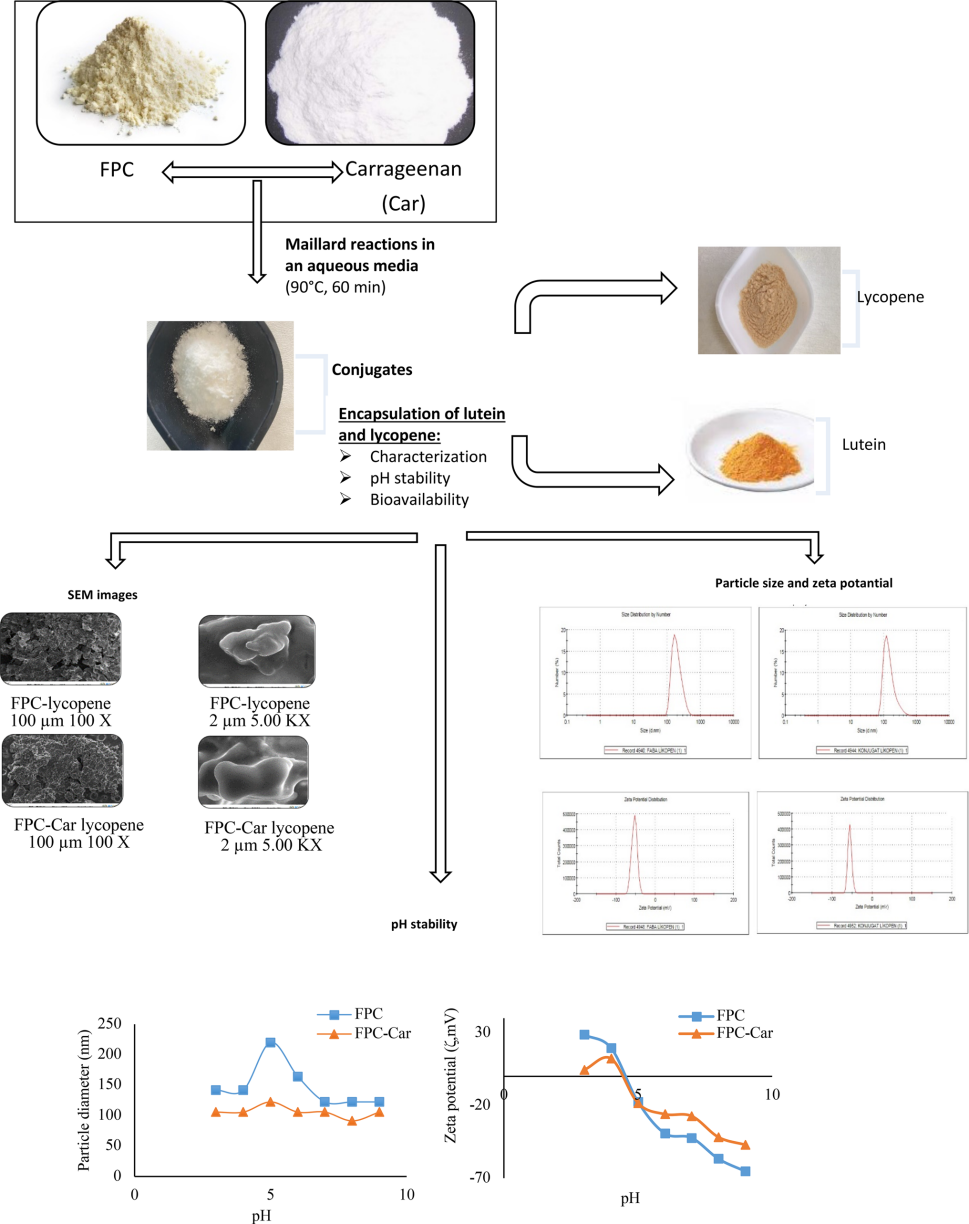

**Supplementary Information:**

The online version contains supplementary material available at 10.1007/s13197-024-05976-4.

## Introduction

Carotenoids are naturally occurring fat-soluble pigments, divided into two groups known as carotenes (*β*-carotene, lycopene) and xanthophylls (lutein and zeaxanthin). Lutein is a natural pigment found in flowers, cereals, fruits, and leafy green vegetables such as, spinach and kale. Lutein has been demonstrated to have a variety of biological effects that can be beneficial, making it an attractive food ingredient for substituting for artificial colors and developing functional foods (Ochoa Becerra et al. 2020). Natural red carotenoid pigment called lycopene can be found in foods such as tomatoes, grapefruit, watermelon, papaya, guava, and other fruits. Lycopene has received a much of interest in recent years due to its role in preventing chronic diseases like atherosclerosis, skin cancer, and prostate cancer. Due to its instability and the chemical alterations brought on by food processing, lutein and lycopene application in the food industry is limited. Processing conditions such as high temperature, presence of oxygen, light, and extreme pH, may affect the integrity of lutein and lycopene into food items. These processing conditions restrict their dissolution in aqueous-based foods and their absorption through gastrointestinal tract remarkably, thus reducing their bioaccessibility. The main challenges to incorporating lutein and lycopene into commercial food products are their low water-solubility, limited chemical stability, and low oral bioaccessibility. By using MRPs as a wall material to encapsulate these carotenoids, it would enable to overcome these difficulties and facilitate their incorporation as a colorant and nutraceutical into functional foods and beverages, such as juices, bakery products, infant formulations, and meat products. Therefore, encapsulation techniques are an effective way to protect against oxidation and degradation by entrapping bioactive chemicals in a carrier. Encapsulation can promote optimized delivery and slows down active components chemical degradation, therefore increases shelf life and bioavailability (Yang et al. 2022). Enhancing the encapsulation of bioactive compounds within nanoemulsions could improve their dispersion within food products, thereby increasing their bioaccessibility in foods (Khanniri et al. 2016; Li et al. 2023). Furthermore, loading tocopherol into oil-in-water nanoemulsions may improve its chemical stability, enhance its antioxidant properties, and increase its bioaccessibility. Studies indicate that tocopherol nanoemulsions with concentrations of 250 and 500 mg/kg were successful in delaying lipid oxidation of fish sausages during refrigerated storage (Feng et al. 2020).

In recent years, there has been a growing interest to protein-polysaccharide conjugates, generated by Maillard reaction, for applications in food, medicine and cosmetic (Augustin et al. 2006). The food and pharmaceutical industries benefit from Maillard reaction products (MRPs) to increase microcapsule stability in the face of extreme conditions such thermal processing, acidic pH, and ionic salt. Therefore, the bioactive chemicals entrapped by MRPs are less prone to stomach digestion, resulting extended its shelf life. Recent developments in encapsulation lead to MRPs as encapsulation agents to encapsulate bioactive materials such as citral (Yang et al. 2022), conjugated linoleic acid (Li et al. 2022), *β*-carotene (Hu et al. 2021), curcumin (He et al. 2021).

Carrageenan is a natural polysaccharide and can be extracted from edible red seaweeds, among which kappa-carrageenan and iota-carrageenan are commonly used in the food industry to improve the functionality and stability of food products. Glycation with carrageenan with various food grade protein has gained attention (Yang et al. 2020). Mao et al. (2018) showed that protective roles of MRPs formed with soy protein isolate-iota carrageenan conjugates on *Bifidobacterium longum*. Seo and Yoo (2021) reported that milk protein isolate could be effectively conjugated with kappa carrageenan under wet heating conditions and milk protein isolate- kappa carrageenan conjugates had great potential as natural emulsifiers and stabilizers in food industry.

Most studies have focused on the effects of reaction conditions and usage as an emulsifier agent, stabilizer, encapsulation agent of MRPs (Yang et al. 2022; Zhong et al. 2021). However, there is still a need for its usage as wall material for the encapsulation of bioactive chemicals and improve their bioaccessibility. Therefore, the main objective of this research was to develop natural lutein and lycopene nanoparticles using protein-polysaccharide MRPs as wall materials and to evaluate their bioaccessibility during intestinal delivery. In this study, FPC-Car conjugates were prepared by wet-Maillard reactions. The encapsulated nanoparticles have been characterized in terms of particle diameter, zeta potential and morphology by using zeta sizer and scanning electron microscopy (SEM). The stability during different pH was also investigated*.* The information obtained from this study provides a new solution for the protection of bioactive chemicals and broadens the choice of MPRs as wall material for encapsulation.

## Material and methods

### Material

κ-Carrageenan (Car) was purchased from Sigma Aldrich. Ingredion Germany GmbH generously provided faba bean protein concentrate (FPC, VITESSENCE^®^ Pulse CT 3602) in support of our study. Disodium hydrogen phosphate, sodium dihydrogen phosphate, potassium bromide, sodium azide, lutein, ɑ-amylase, pepsin, bile extract, pancreatic enzymes, lipase, glycerol, ethyl acetate and EDTA were purchased from Sigma Aldrich and Merck. Lycopene was purchased from Ark Farm. All chemicals were analytical grade.

### Production of wall material

The encapsulation wall material was formed by conjugation of faba bean protein concentrate (FPC) with Car by Maillard reaction in an aqueous media. The conjugation procedure, 1% FPC (w/w) and 2% Car (w/w) were dissolved in 100 mL phosphate buffer solution (0.2 M, pH 7) and then stirred by a magnetic stirrer for 2 h, followed by gently stirring overnight at 4 °C to completely hydrate. The conjugation ratios determined according to our previous study (Dursun Capar and Yalcin 2021). The pH value of the solution was adjusted to 7.0 by adding 0.1 N HCl or 0.1 N NaOH. The solution was heated at 90 °C for 60 min. Immediate ice bath was used for the cooling the solution at the end of the heating time. Then, the solution was centrifuged (Hitachi High-Speed Refrigerated Centrifuge CR22N, Japan) for 15 min at 30.000 g at 4 °C. The supernatant part was separated and freeze-dried (CHRIST, Alpha 2–4 Lsc Plus, Germany).

### Conjugation efficiency and conjugation yield

Conjugation yield and efficiency was determined according to Markman and Livney (2012). Briefly, OPA (*o*-Phthaldialdehyde) assay was carried out for determining conjugation efficiency and it is calculated according to the following formula:

$${\text{CE}}\left( \% \right) = \left( {{ }1 - \frac{{{\text{amine }}\;{\text{groups}}\;{\text{after}}\;{\text{ conjugation}}}}{{{\text{amine }}\;{\text{groups}}\;{\text{ before}}\;{\text{ conjugation}}}}} \right) \times 100$$

Conjugation yield was calculated according to the formula given below:

$${\text{CY}} \left( \% \right) = \left( {\frac{{{\text{protein }}\;{\text{in }}\;{\text{supernatant}} \left( {\frac{{{\text{mg}}}}{{\text{L}}}} \right)}}{{{\text{protein}}\;{\text{ in }}\;{\text{suspension}}\left( {\frac{{{\text{mg}}}}{{\text{L}}}} \right)}}} \right) \times 100.$$

### Protein solubility

The protein solubility of the conjugated biopolymer was determined according to Bradford assay with slight modifications. Briefly, the samples dissolved in buffer solutions (2 M Sodium phosphate, pH 7) (100 µL/mL) and agitated for 30 min at room temperature. 40 µL sample and 200 µL dye solution (Coomassie Brilliant Blue dissolved in 95% ethanol) were blended in a 96-microwell plate. The plate was left to stir for 1 min, then stand for 10 min and the absorbance was measured at 620 nm (Microreader, Multiscan FC, Thermo Fisher, USA). 40 µL buffer solution and 200 µL dye solution was prepared for blank. The protein solubility of the samples was subsequently calculated using a calibration curve prepared using bovine serum albumin (BSA) concentrations ranging from 0 to 2.0 mg/mL. The protein amounts of unprocessed protein samples were determined, and solubility was calculated as %.

### Encapsulation and encapsulation yield

Native FPC and FPC-Car conjugates (1%, w/w) was dissolved in ultrapure water at the pH of 7.0, and sodium azide was added to inhibit microbial growth. The mixture was stirred at room temperature for 2 h. Gum Arabic (1%, w/w) was dissolved in pure water and waited at 4 °C for one night to completely hydrate. Then, lutein and lycopene solutions (0.1%, w/w) were prepared in ethyl acetate, separately. Lutein loaded nanoparticles were prepared according to the method described by Tan and Nakajima (2005) with slight modifications. Before preparing the protein solution, 100 mL of water were saturated with 8.3 g of ethyl acetate to avoid lutein from precipitating during homogenization. At a ratio of 1:9, saturated protein ethyl acetate solution and lutein-containing solution were combined. All biopolymers were combined and then, 3% (w/w) corn oil and 1% (w/w) Tween 20 were added to the mixture. The loaded concentration of the lutein was 1% (m_lutein_/m_lipid_, w/w). Lutein containing ethyl acetate solution was combined with biopolymer solution and homogenized for 2 min at 10,000 rpm by an Ultra-Turrax (IKA, Germany) to form a coarse emulsion. Ultrasound (UP 400S, Hielscher, Germany) was applied (amplitude:60Watt, cycle:1) to the resulting emulsion to form nanoemulsion (particle size < 500 nm). After homogenization, ethyl acetate was removed by using a Rotary Evaporator at 30 °C for 30 min. Zeta potential and particle size analyses were performed for biopolymer nanoparticles, and then the nanoparticle solution was lyophilized (CHRIST, Alpha 2–4 Lsc Plus, Germany). This method was applied to lycopene in the same way.

Lutein and lycopene content and encapsulation efficiency (EE) of the nanoparticles were evaluated according to Tan et al. (2016). The content of free lutein or lycopene suspended in aqueous phase was extracted with n-hexane. For this purpose, 0.3 g sample dissolved in 3 mL n-hexane and stirred for 3 min. Then, the mixture centrifuged at 2000 rpm for 5 min and the supernatant was taken. Same procedure repeated twice, and all supernatants were collected. The total volume diluted to 10 mL with n-hexane. The free amount of lutein or lycopene determined spectrophotometrically at 445 and 472 nm, respectively. The experiment was carried out in triplicates. The total amount is referred as the amount of lycopene or lutein added at the initial stage of experiment. Lutein or lycopene encapsulation efficiency (EE) and loading capacity (LC) were calculated following formula;

$${\text{EE }}({\text{\% }}){ } = \frac{{\left( {{\text{total }}\;{\text{lutein }}\;{\text{or }}\;{\text{lycopene}} - {\text{free}}\;{\text{ lutein}}\;{\text{ or}}\;{\text{ lycopene}}} \right)}}{{{\text{total }}\;{\text{lutein }}\;{\text{or }}\;{\text{lycopene}}}} \times 100$$

$${\text{LC }}\left( {\text{\% }} \right) = \frac{{\left( {{\text{total }}\;{\text{lutein }}\;{\text{or }}\;{\text{lycopene}} - {\text{free}}\;{\text{ lutein }}\;{\text{or }}\;{\text{lycopene}}} \right)}}{{{\text{total }}\;{\text{amount }}\;{\text{of }}\;{\text{nanoparticle}}}} \times 100.$$

### Nanoparticle characterization (particle size, zeta potential and SEM images)

The characteristic properties of lutein and lycopene loaded nanoparticles were determined by using the Zetasizer ZS90 device (Malvern Instruments, UK), measuring the average particle diameter, polydispersity index (PDI), distribution diameter and zeta potentials of the particles.

Scanning electron microscopy measurements were conducted by using SEM LEO 440 Stereoscan which is equipped with an EDX and WDX 600i X-Ray spectrophotometer. 8 different images (500X, 5KX, and 10KX) were taken. In order to prevent multi-scattering effects, nanoparticles were diluted 100 times with ultra-pure water and the pH has been adjusting to 7. The measurements were conducted for 3 times.

### In-vitro analysis and stability

A dynamic in-vitro gastrointestinal model was used to study the effect of encapsulated lutein and lycopene on body bioaccessibility. The gastrointestinal model was carried out according to the method described by Minekus, et al. (2014). The stock solutions of the simulated digestion fluids (simulated salivary fluids (SSF), simulated gastric fluids (SGF), simulated intestinal fluid (SIF)) used in the bioaccessibility analysis were prepared according to Supplementary document Table 1. All reagents warmed to 37 °C before the analysis.

**Table 1:** Particle diameter and zeta potential of lycopene or lutein loaded FPC-Car conjugates and FPC

Wall material	Compound	Particle diameter (nm)	PDI	Zeta potential (mV)
FPC	Lycopene	200.74^a*^	0.30^b^	-52.8^b^
FPC-Car conjugate	Lycopene	154.09^b*^	0.29^a^	-55.5^a^
FPC	Lutein	71.49^a^	0.27^a^	-48.2^b*^
FPC-Car conjugate	Lutein	66.60^b^	0.24^b^	-63.9^a*^

Different small letters in the same column show that there is a statistically significant difference between the data (*p* < 0.05, *p < 0.01)

*PDI* polydispersity index

#### Simulated oral phase

For the simulated oral phase, 5 mL of liquid food mixed with 3.5 mL of SSF. All solutions were pre-heated to 37 °C before use. 0.5 mL salivary α-amylase solution (1500 U/mL) made up in SSF stock solution (α-amylase from human saliva, Type IX-A, lyophilized powder, 1000–3000 units/mg protein, Sigma) is added. Then, 25 μL 0.3 M CaCl_2_ and 975 μL of water were added. The pH of the mixture is set to 7 and stirred at 37 °C for 2 min with 100 rpm interpreting speed.

#### Simulated gastric phase

Simulated gastric fluid stock solution (SGFSS) was prepared by dissolving 2 g of NaCl and 7 mL of HCl (37%) in 1 L of double distilled water. Simulated gastric fluid work solution (SGFWS) was prepared by mixing 20 mL of SGFSS and 0.064 g of pepsin (amounts are per sample) 45 min before running the gastric phase. The pH of the oral phase sample (5 mL) was adjusted to 3 using HCl (2 M) and incubated at 37 °C for 2 h with continuous agitation at 100 rpm.

#### Simulated small intestinal phase

5 mL of gastric chyme was placed in a water bath at 37 °C and the pH was set to the 7 using NaOH. Then, 4 mL SIF was added. Afterwards, bile extract (5 mg/mL), pancreatin (0.9 mg/mL) and 10 µL CaCl_2_ (37 mg/mL) incorporated into the mixture. The pH was re-adjusted to 7 using NaOH and incubated at 37 °C for 2 h with continuous agitation at 100 rpm. At the end of the time, the pH was adjusted to 3.

#### Bioaccessibility

The bioaccessibility of lutein and lycopene was evaluated after the samples had passed through the simulated small intestine phase of the gastrointestinal model.

The samples (10 mL) were centrifuged at 4000 rpm for 40 min at room temperature (Hitachi High-Speed Refrigerated Centrifuge CR22N, Japonya). After centrifugation, the samples separated into a sediment phase at the bottom and a clear micelle phase at the top. The intestinal phase and the micelle phase were dissolved in dimethyl sulfoxide and absorbances read at 460 nm for lutein and 472 nm lycopene. The bioaccessibility (BA) was determined using the following equation:

$$BA \left( \% \right) = \frac{C_M }{{C_R }} \times 100$$

where C_M_, is the lutein or lycopene concentration in the micelle phase and C_R_ is the concentration of lutein or lycopene in the entire intestinal phase. The samples without lutein or lycopene were used as blanks. The concentration of lutein or lycopene was determined from absorbance measurements (460 nm for lutein, 472 nm for lycopene) made using a UV–visible spectrophotometer (UV–VIS, Agilent Technologies, USA). A calibration curve was prepared for lutein and lycopene.

#### pH stability

pH of the nanoparticle solutions was adjusted from 3 to 8 using HCl and NaOH and left at room temperature for 4 h before the particle characterization analysis. Average particle diameter, diameter distribution and zeta potentials were measured using the Zetasizer ZS90 instrument (Malvern Instruments, UK).

### Statistical analysis

All the experiments carried out in triplicates and the data were reported as means. Tukey’s multiple range test was applied to evaluate the differences among the samples with 95% confidence level (Minitab 17.0).

## Results and discussion

### Conjugation efficiency, conjugation yield and protein solubility

Conjugation efficiency (CE) of the FPC-Car conjugated sample was found as 25.77%. Also, conjugation yield (CY) was found as 94.32%. Conjugation of protein and polysaccharide is affected many factors such as time, temperature, pH, protein or polysaccharide type, weight ratio and others. The formation of conjugate was confirmed by our previous study (Dursun Capar and Yalcin 2021). The highest ratio for conjugation of FPC and Car were found as 1:2 in our previous study. Therefore, the most optimal weight ratio was 1:2 in for this study. Functional characteristics like emulsion stability, solubility, heat stability, and surface hydrophobicity are significantly influenced by Maillard conjugation (Cui et al. 2020). Protein solubility (PS) of the conjugated sample was found as 42.59%. Pea protein-gum Arabic conjugates (Zha et al. 2019a, b) and pea protein-pectin conjugates (Tamnak et al. 2016) have been employed in different studies. It has been concluded that conjugation can increase the protein's functionality (solubility, emulsification, and thermal stability), which may increase its acceptability and efficiency.

### Nanoparticle characterization

**Fig. 1:**
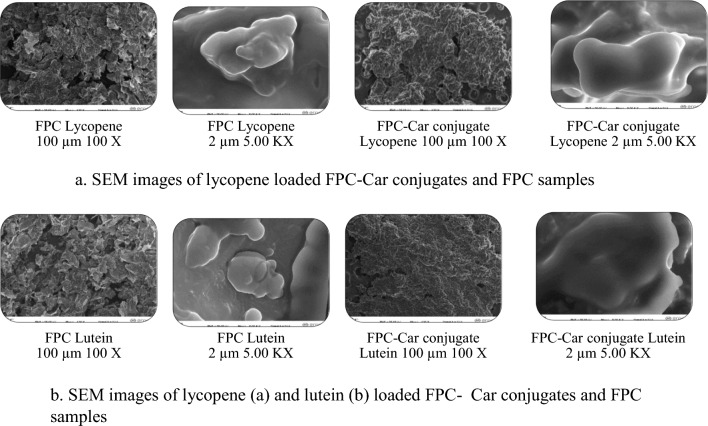
SEM images of lycopene (**a**) and lutein (**b**) loaded FPC-Car conjugates and FPC samples

**Fig. 2:**
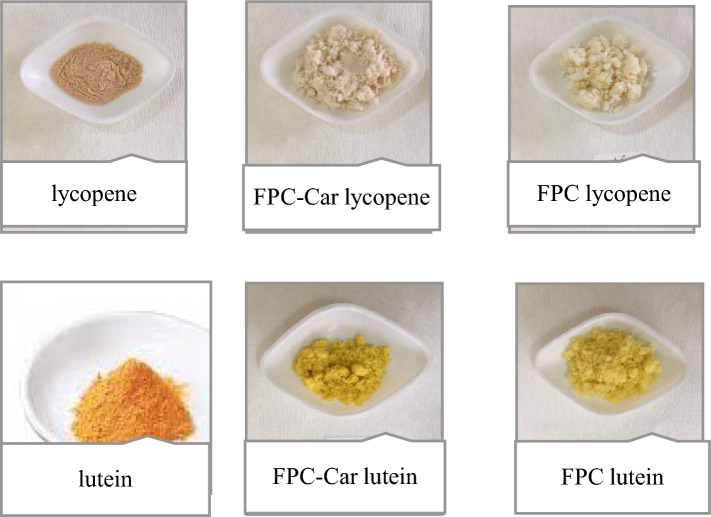
Pictures of encapsulated lutein and lycopene with FPC-Car conjugate and FPC

Particle size and zeta potentials of the lycopene or lutein loaded FPC-Car and FPC nanoparticles were shown in supplementary document Figs. 1a, b and 2a, b. Zeta potentials of lycopene loaded FPC-Car conjugate and FPC samples were found as − 55.5 and − 52.8 mV, respectively. Zeta potentials of lutein loaded FPC-Car conjugate and FPC samples were found as − 48.2 and − 63.9 mV, respectively (Table 1). Zeta potential of plain FPC were found as − 35.0 mV. Zeta potential measurements showed that nanoparticles generated by FPC-Car conjugate are strongly negative. Furthermore, results revealed that Car was absorbed by creating a pair of layers on the droplets FPC. The zeta potential measurements showed negative values, which indicated a strong repulsion between them. This finding was consistent with the zeta potential value observed in tea water in soluble protein-carrageenan mixture (Ren et al. 2021). Typically, a magnitude of zeta potential varies from + 100 to − 100 mV (Abbasi et al. 2019). A higher zeta potential (positive or negative) signifies a stable system (Abbasi et al. 2019). Zeta potential determines the interaction energy between each of the colloidal particles, which in turn determines the stability of colloidal particles against coagulation or aggregation (Assadpour et al. 2020a). High electrostatic charge prevents aggregation of the droplets due to the electrostatic repulsion between particles (Assadpour et al. 2020b). Zeta potential of the systems depends on the charge on the actual particle and the charge related to any ions that move along with the particle in the electric field (Surh et al. 2006). Therefore, the negative charge could be related to the anionic components of FPC-Car conjugate. It is reported that droplets coated by multi-layered interfacial membranes often have improved the stability to environmental stress than those stabilized by one-layered membranes due to the increase in rheology and interfacial thickness (McClements 2004). A multi-layered emulsion prepared by polysaccharides and proteins may be related either through electrostatic interactions (Abbasi et al. 2019).

Regardless of the composition of the stabilizing agent, the zeta potential mostly depends on the medium's pH. Unfortunately, the literature reports no specific value for zeta-potential measurement, which is frequently expressed as “all measurements have been performed after adequate dilution of an aliquot of the suspension in water”. It's difficult to predict overall behavior when pH and salinity of the medium are unknown. However, because of the high-energy barrier between particles, zeta-potential values lower than − 10 mV (typically between − 25 and − 30 mV) are recorded, providing strong colloidal stability to be predicted (Mora-Huertas et al. 2010).

Droplet size measurements of lutein or lycopene loaded nanoparticles obtained by dynamic light scattering (DLS) are depicted in supplementary document Figs. 1a, b and 2a, b. It is apparent from Table 1 that the average particle diameter of nanoparticles coated with FPC was found significantly higher than FPC-Car conjugate (*p* < 0.05). DLS results revealed that lycopene nanoparticles coated by FPC was found as 200.74 nm, whereas 145.09 nm for the FPC-Car conjugate. The mean particle diameters were found as 71.49 nm and 66.60 nm for lutein loaded nanoparticles coated by FPC and FPC-Car conjugate, respectively. In contrast to FPC, the average particle size of FPC-Car conjugates was noticeably smaller, and the absolute value of zeta potential was significantly greater. These findings suggested that glycation modification increased the electrostatic repulsion between the droplets, while the introduction of sugar chains also enhanced the thickness of the interface film, thereby contributing to the stability of the droplets (Liu et al. 2018; Bu et al. 2023).

Another important factor that indicates the width or spread of the particle size distribution is the polydispersity index (PDI). This value may vary from 0 to 1, where values less than 0.4 indicate greater stability for a nanodelivery/colloidal system. According to Table 1, the PDI values of the samples were smaller than 0.4, which implies a monomodal particle size. DLS results displayed that samples coated with FPC-Car conjugates had relatively homogeneous distribution and the average particle size was less than 1000 nm. The stability of the FPC-Car conjugate could be associated with size distribution of the small particles in the system. The average particle diameters of the obtained nanoparticles concur well with the previous studies (Gumus et al. 2017).

The surface morphology of the lycopene or lutein nanoparticles produced using FPC and FPC-Car conjugates were observed using scanning electron microscope. It is apparent from Fig. 1a and b that nanoparticles produced with FPC-Car conjugate as a wall material were spherical. There were no fractures or fissures on the surface, revealing that wall materials provided better protection and retention for core materials (Jia et al. 2020). The formation of some typical depressions may be caused by the shrinkage of nanoparticles during lyophilization process. The formation of the smooth and less concave-convex surface could be due to better coating properties of FPC-Car conjugates. The high emulsification properties of the conjugates may also have a significant effect on the morphological properties of nanoparticles. Hence, encapsulating of lutein or lycopene using conjugates as covering materials improve their stability.

In a study, whey protein isolate-xylo-oligosaccharide conjugates (reaction temperature 90 °C, heating time 3 h and pH 9) were used as a wall material to encapsulate lycopene. According to SEM images results, lycopene microcapsules were found as spherical and the whole surface was continuous with some typical concavities (Jia et al. 2020).

In another study, whey protein isolate and galactose conjugates were used as coating materials to form beta carotene microcapsules. WPI-galactose mixture produced a rough and more concave-convex surface whereas the microparticles prepared with the WPI-galactose conjugates displayed a smooth and less concave-convex surface (Jiang et al. 2017).

### Encapsulation yield

Encapsulation yield generally is defined as the percentage of capsulated molecule in nanoparticles according to the total amount used (Jafari 2017). The encapsulation yield and loading capacity of lycopene or lutein nanoparticles prepared with FPC and FPC-Car conjugate are given in Table 2. The pictures of encapsulated lutein and lycopene with FPC-Car conjugate and FPC were given in Fig. 2. According to Table 2, 77.58% of lycopene was coated with FPC and only 22.42% remained without coated. 82.69% of lycopene was coated with FPC-Car conjugate and 17.31% remained without coated. Encapsulation yield of lutein nanoparticles coated with FPC-Car conjugate was found to be the highest with 93.07%, while the yield of plain FPC was 89.51%. The encapsulation yield of lycopene or lutein coated with FPC-Car conjugate was found to be higher than plain FPC. Our findings consistent with Bu et al. (2023)’s findings reported that glycated soy protein isolate exhibited higher carotenoid encapsulation efficiency (94%) compared to soy protein isolate.

**Table 2:** Encapsulation yield and loading capacity of lycopene or lutein nanoparticles

Coating material	Coated compound	Encapsulation yield (%)	Loading capacity (%)
FPC	Lycopene	77.58^b*^	7.32^b*^
FPC-Car	Lycopene	82.69^a*^	9.20^a*^
FPC	Lutein	89.51^b^	0.49^a^
FPC-Car	Lutein	93.07^a^	0.5^a^

Different small letters in the same column show that there is a statistically significant difference between the data (*p* < 0.05, *p < 0.01)

The encapsulation yield of the bioactive compounds varies depending on the size or surface area of the nanoparticle, type and composition of the wall material, emulsifying agent and sonication time (Ghorbanzade et al. 2017). In general, it has been reported that reducing the size of emulsion particles, which increases stability, results in improved encapsulation effectiveness and hence enhanced bioactive material preservation (Abbasi et al. 2019). However, in different studies, it has been reported that the encapsulation yield is not related to the emulsion particle size and this difference is due to the use of polymer matrices with different protection properties (Mora-Huertas et al. 2010). According to the results of the particle size analysis given in Table 1, the particle diameters of the samples covered with FPC-Car conjugate are lower than the samples covered with FPC. It is thought that the decrease in particle size increased the encapsulation yield.

The loading capacities of biopolymer nanoparticles are given in Table 2. The loading capacities of FPC-Car conjugates were higher than FPC. The highest loading capacity was obtained in lycopene loaded FPC- Car conjugate with 9.20%. The lowest loading capacity was obtained in FPC lutein loaded sample with 0.49%.

Different factors affecting the encapsulation yield have been reported (Mora-Huertas et al. 2010). The chemical nature of the coated material and polarity affect encapsulation yield. In this way, although hydrophilic components have a maximum encapsulation yield of 10%, lipophilic molecules have a yield of more than 70% (Stella et al. 2007). In this study, encapsulated lutein or lycopene are lipophilic and the yield of the encapsulation is compatible with the literature.

### pH stability

**Fig. 3:**
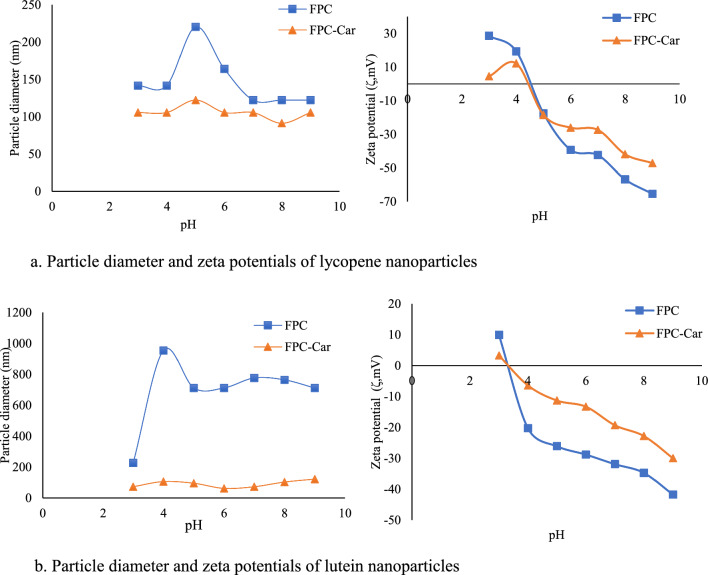
Particle diameter and zeta potentials of lycopene (**a**) and lutein (**b**) nanoparticles

The average particle size and zeta potentials of the nanoparticles during the pH 3 to 9 were examined. According to changes of pH, particle size and zeta potential changes are given in Fig. 3a for lycopene nanoparticles and Fig. 3b for lutein nanoparticles.

The diameter of lycopene nanoparticles coated with FPC remained constant between pH 3–4 and 7–9 but increased to 220 nm at pH 3–4 (Fig. 3a). Even though the diameter of lutein nanoparticles coated with FPC remains steady between pH 5 and 9, increased to 953 nm at pH 3 (Fig. 3b). Nanoparticles are aggregating around the isoelectric point, based on the particle size of the proteins. Under the same pH conditions, FPC-Car conjugates and lutein or lycopene loaded FPC-Car nanoparticles showed no significant changes in particle diameter (*p* > 0.05). The lack of aggregation and zeta potential findings confirm the importance of carrageenan’s steric hindrance at the interface in preventing nanoparticle flocculation or aggregation, as well as separation of the protein surface from the continuous media (Lesmes and McClements 2012). As indicated by Yi et al. (2014), conjugate based nanoparticles are more stable than native proteins under variable pH conditions. Our results indicate that covalent interactions exist between FPC-Car, where the negatively charged sulphate groups on Car could interact with the positively charged amino groups on proteins at a pH above the isoelectric point (Lu et al. 2023). Protein/polysaccharide complexes are non-covalent bonds that form due to the attractive electrostatic interaction between anionic polysaccharides and positively charged proteins (Evans et al. 2013). Electrostatic interaction can enhance the heat stability of emulsions by creating a homogenous biopolymer layer on the surface of oil droplets. However, these complexes are sensitive to environmental conditions because the protein–polysaccharide interaction is influenced by solvent conditions such as pH and ionic strength (Doost et al. 2019). In contrast, molecular complexes are covalent bonds that can occur spontaneously during the early stages of the Maillard reaction (Nooshkam and Varidi 2020). The polysaccharide moieties of molecular complexes provide greater layer thickness on the droplet surfaces, which can improve stability due to the strong steric repulsion between droplets (Zhu et al. 2022). Additionally, the thickness and steric stabilization effects of molecular complexes-stabilized droplets can offset the electrostatic screening effects, which can be stable at high salt concentrations and under acidic pH (Akhtar and Ding 2017). Protein-based nanoparticles can flocculate or congregate around their isoelectric point (pI), (Lesmes and McClements 2012), and cause an increase in mean particle diameter at this point. This behaviour limits their use in food systems, particularly in slightly acidic beverages like juice, yogurt, and functional beverages. To solve this problem, large carbohydrates were utilized to glycate via the Maillard reaction, reducing the quantity of basic groups and increasing steric hindrance.

### Bioaccessibility

The bioaccessibility of the lutein or lycopene loaded biopolymer nanoparticles were presented in Table 3. The bioaccessibility of the lutein or lycopene samples encapsulated by FPC were found as higher than FPC-Car conjugates. It was found that 82.13% for FPC encapsulated lycopene whereas more than 10.9% compared FPC-Car conjugate. These values are appreciably higher than other studies (Jia et al. 2020). The bioaccessibility of lutein loaded FPC and FPC-Car conjugate samples were found as 61.15% and 38.70%, respectively. The bioaccessibility of the lycopene samples was found higher compared lutein samples (Table 3). This may be resulted from the better dissolution of lycopene in micelles, and as a result gives higher bioaccessibility than lutein. Furthermore, the reason of the lower bioaccessibility of the conjugates can be caused by the fact that they remain more irregularly in the simulated gastric environment than native FPC.

**Table 3:** Bioaccessibility of lutein and lycopene loaded nanoparticles

Coating material	Coated compound	Bioaccessibility (%)
FPC	Lycopene	82.13^a^
FPC-Car	Lycopene	71.23^b^
FPC	Lutein	61.15^a^
FPC-Car	Lutein	38.70^b^

Different small letters in the same column show that there is a statistically significant difference between the data (*p* < 0.05, *p < 0.01)

The bioaccessibility is the portion of the substance that was soluble in the micelle phase and so accessible to the small intestinal epithelium for absorption. The bioaccessibility of carotenoids is affected many factors such as type of carotenoid, food matrix composition, amount of carotenoid ingested, genetic factors and the interaction of the above factors (Boonlao et al. 2022).

Jia et al. (2020) indicated the free lycopene and whey protein conjugate (WPC)’s bioaccessibility as 16 ± 3% and 60 ± 4%, respectively. A recent study of the literature on lutein encapsulation found that bioaccessibility was 14.6% and 19.3% in the mixture and bovine serum albumin-fucoidan conjugate, respectively. Although, they concluded WPC improved the bioaccessibility, our results do not support previous research in this area. In fact, unlike what was previously thought, we found that conjugation did not improve the lutein and lycopene bioaccessibility. However, the bioaccessibility of conjugates found higher compared to other studies (Kim and Shin 2021).

## Conclusion

Lutein and lycopene loaded nanoparticles were successfully prepared via wet-Maillard reactions products of FPC-Car. In this study, FPC-Car (1:2) was heated at 90 °C for 60 min to form MRP conjugates. The conjugation yield and efficiency were found as 94.32% and 25.77%, respectively. Meanwhile, encapsulation yield of nanoparticles coated with MRPs was significantly improved. SEM images showed that lutein and lycopene nanoparticles were spherical and the whole surface was continuous. Stability study was carried out under different pH values. Nanoparticles produced from FPC-Car conjugates were more stable than FPC. There have been no major changes observed in particle diameters under the same pH conditions of lutein and lycopene loaded FPC-Car conjugates. Although the bioaccessibility of conjugates were found lower compared to native FPC, it was found higher compared to others’ findings. According to this study, conjugates produced from a glycated plant protein (FPC) with Car can be effectively used as an encapsulation wall material to deliver carotenoids. As a result, FPC-Car conjugates may be an alternative for the formation of functional beverages as well as other nutraceutical products, such as juices, bakery products, infant formulations, and meat products. Furthermore, since the FPC-Car conjugates did not increase the bioaccessibility of lutein and lycopene during digestion, it is suggested to carry out additional research on this area.

## Supplementary Information

Below is the link to the electronic supplementary material.

Supplementary file1 (DOCX 923 kb)

## Data Availability

The data that support the findings of this study are available on request from the corresponding author.
