# Supplementary material for: Conjugation prepared by wet-Maillard reactions improves the stability and properties of lutein and lycopene loaded nanoparticles
Source: J Food Sci Technol. 2024 Apr 7;61(10):2008–19. doi: 10.1007/s13197-024-05976-4 (PMC11401807; doi:10.1007/s13197-024-05976-4)
Supplement: Supplementary file 1 — Supplementary file1 (DOCX 923 kb) [file 13197_2024_5976_MOESM1_ESM.docx]

**Supplementry document**

SD Table 1. Simulated digestive fluids concentrations of stock solutions

Table 1. Simulated digestive fluids concentrations of stock solutions [20]

|  |  | | SSF |  | SGF |  | SIF |  |
| --- | --- | --- | --- | --- | --- | --- | --- | --- |
|  |  | | pH 7 |  | pH 3 |  | pH 7 |  |
| Components | Stock concentration | | Stock volume | Stock concentration | Stock volume | Stock concentration | Stock volume | Stock concentration |
|  | g/L^-1^ | mol/L^-1^ | mol/L^-1^ | mmol L^-1^ | mL | mmol L^-1^ | mL | mmol L^-1^ |
| KCl | 37.3 | 0.5 | 15.1 | 15.1 | 6.9 | 6.9 | 6.8 | 6.8 |
| KH_2_PO_4_ | 68 | 0.5 | 3.7 | 3.7 | 0.9 | 0.9 | 0.8 | 0.8 |
| NaHCO_3_ | 84 | 1 | 6.8 | 13.6 | 12.5 | 25 | 42.5 | 85 |
| NaCl | 117 | 2 | - | - | 11.8 | 47.2 | 9.6 | 38.4 |
| MgCl_2_(H_2_O)_6_ | 30.5 | 0.15 | 0.5 | 0.15 | 0.4 | 0.1 | 1.1 | 0.33 |
| (NH_4_)_2_CO_3_ | 48 | 0.5 | 0.06 | 0.06 | 0.5 | 0.5 | - | - |

SD Figure 1. Particle size (a-b) and zeta potential (c-d) of lycopene loaded FPC-Car conjugates and FPC

FPC lycopene

FPC- Car conjugate lycopene

FPC lycopene

FPC- Car conjugate lycopene


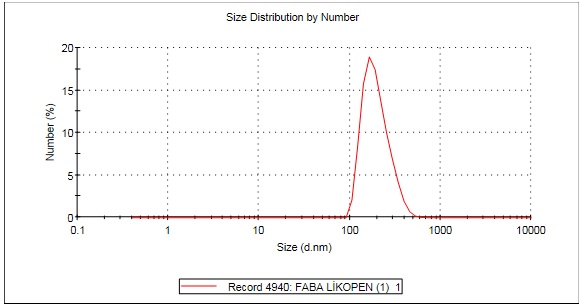


a


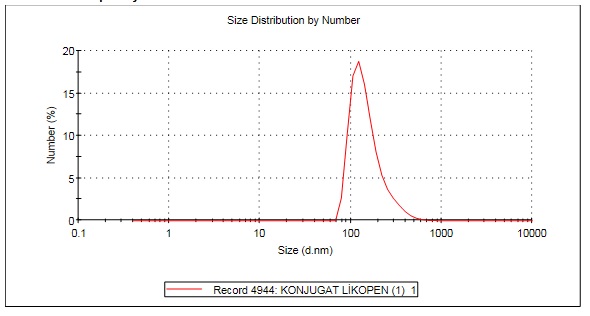


b


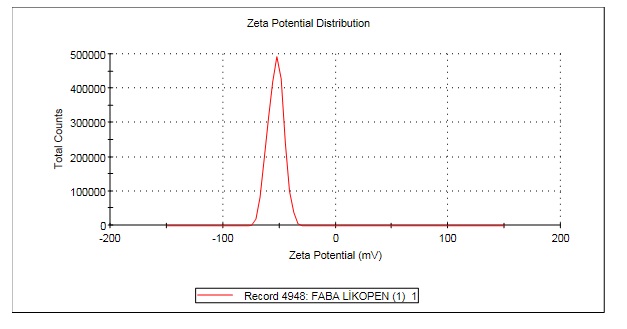


c


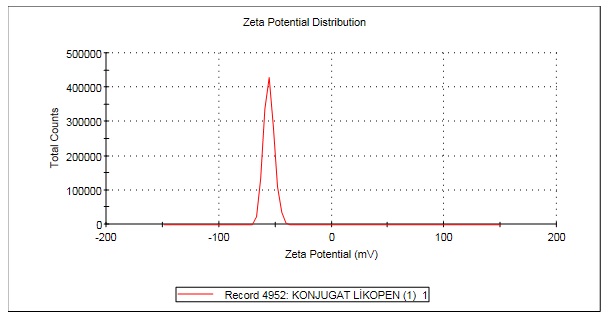


d

SD Figure 2. Particle size (a-b) and zeta potential (c-d) of lutein loaded FPC-Car conjugates and FPC

FPC lutein

FPC-Car conjugate lutein

FPC-Car conjugate lutein

FPC lutein


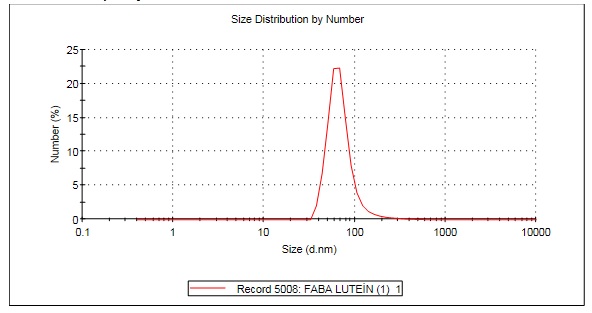


a


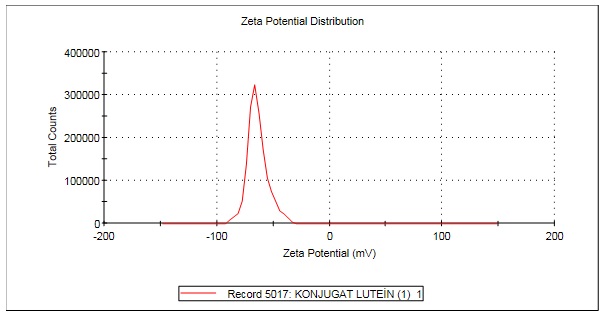


d


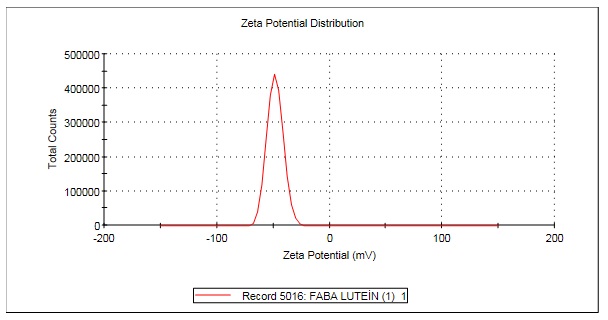


c


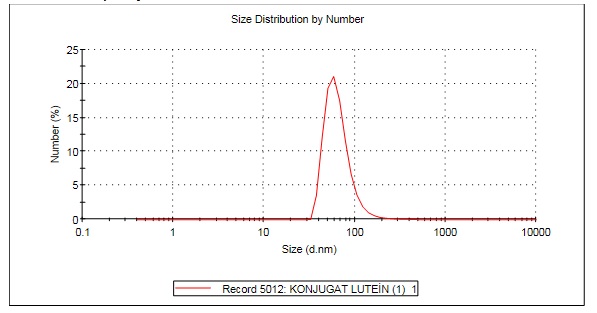


b
